# A pilot study: Salivary human herpesvirus‐6 and human herpesvirus‐7 responses to different types of acute exercise in healthy young men

**DOI:** 10.14814/phy2.70697

**Published:** 2025-12-19

**Authors:** Masataka Uchida, Chihiro Kojima, Takeshi Hashimoto, Kohei Watanabe, Taichi Nishikawa, Tadao Isaka, Motoyuki Iemitsu

**Affiliations:** ^1^ Research Organization of Science and Technology Ritsumeikan University Kusatsu Japan; ^2^ Institute of Advanced Research for Sport and Health Science Ritsumeikan University Kusatsu Shiga Japan; ^3^ Faculty of Sport and Health Science Ritsumeikan University Kusatsu Shiga Japan; ^4^ Laboratory of Neuromuscular Biomechanics, School of Health and Sport Sciences Chukyo University Toyota Japan; ^5^ Ritsumeikan Global Innovation Research Organization Ritsumeikan University Kusatsu Japan

**Keywords:** human herpesvirus‐6, interval exercise, maximal voluntary contraction, physical fatigue

## Abstract

This study examined the effects of different types of acute exercise on salivary expression levels of human herpesvirus 6 (HHV‐6) and 7 (HHV‐7). In a randomized crossover trial, 11 healthy untrained men performed a continuous exercise (CE) trial (20 min at 70% $\dot{V}O$_2_max) and an interval exercise (IE) trial (20‐min cycling: five sets of 2‐min at 50% $\dot{V}O$_2_max and 2‐min at 90% $\dot{V}O$_2_max). Salivary HHV‐6 and HHV‐7 DNA expression was measured using real‐time PCR, and maximum voluntary contraction (MVC) of knee extensors was assessed after saliva collection. Salivary HHV‐6 expression increased at post‐0 min (*p* < 0.001) and post‐30 min (*p* = 0.002) in IE compared with pre, and was higher in IE than CE at both time points (*p* = 0.002, *p* = 0.048 respectively), but salivary HHV‐7 expression did not change between trials. Changes in serum IL‐6 and blood lactate levels were significantly higher in IE than in CE at post (*p* < 0.001). The sum of time‐dependent changes in MVC was significantly lower in IE than CE (*p* = 0.016). Change in salivary HHV‐6 from pre to post‐24 h was negatively correlated with change in MVC (*r*_s_ = −0.349, *p* = 0.047). These results suggest that IE may increase salivary HHV‐6 expression and that changes in salivary HHV‐6 may reflect objective physical fatigue after acute IE.

## 1 INTRODUCTION

Exercise induces physical fatigue, typically resolving within a few hours to a few days (Meeusen et al., 2013). However, inadequate recovery may lead to “long‐term maladaptation” (Meeusen et al., 2013), resulting in long‐term performance decline, overreaching, overtraining syndrome, and chronic fatigue syndrome (Meeusen et al., 2013). Therefore, fatigue management after exercise is crucial for daily exercise training, necessitating an objective assessment of physical fatigue.

Traditionally, exercise‐induced fatigue has been evaluated using subjective physical fatigue scales, such as psychological questionnaires and visual analog scale (VAS), and objective fatigue scales, such as biochemical, hormonal, and immunological parameters, and autonomic nervous system balance (Bestwick‐Stevenson et al., 2022). Additionally, traditional evaluation scales of objective physical fatigue are influenced by psychological stressors, such as motivation and life events, making it difficult to accurately assess fatigue (Aoki et al., 2016). Furthermore, blood, urine, and saliva samples were used to measure biochemical, hormonal, and immunological parameters as evaluation scales for objective physical fatigue. Saliva can be easily sampled noninvasively. As biomarkers, salivary cortisol and amylase levels have been studied as objective indicators of post‐exercise fatigue (Bestwick‐Stevenson et al., 2022; Meeusen et al., 2013). However, the use of these markers remains controversial owing to the influence of circadian rhythms and menstrual cycles, and their application as markers remains controversial (Chida & Steptoe, 2009; Crewther et al., 2008; Meeusen et al., 2013).

Human herpesvirus (HHV)‐6 and HHV‐7, which cause latent infections (Agut et al., 2017) have been detected in saliva and shown to transiently increase during high‐intensity military training, decreasing after detraining (Aoki et al., 2016). Additionally, salivary HHV‐6 and HHV‐7 levels respond to fatigue induced by various types of exercise training programs for 3 weeks in baseball and rugby university players (Tamai, Sone, et al., 2022). In a 3‐day judo training camp, salivary HHV‐6 and HHV‐7 levels increased after the training camp (Tamai, Hiraoka, et al., 2022). However, these observational studies did not quantitatively control exercise stress, leaving it unclear whether salivary HHV‐6 and HHV‐7 levels respond to quantitatively defined physical stress. Furthermore, although salivary HHV‐6 and HHV‐7 levels have been shown to change in response to fatigue by chronic exercise training, their effects on acute physical stress remain unknown. Several studies have shown that HHV‐6 reactivation is preceded by increases in systemic IL‐6 concentrations (Fujita et al., 2008; Yoshikawa et al., 2006). In healthy men, interval exercise (IE) increased IL‐6 production more than continuous exercise (CE) (Leggate et al., 2010). Therefore, reactivation of HHV‐6 in the saliva may be more easily triggered by IE than CE. To clarify the changes in salivary HHV‐6 and HHV‐7 levels caused by acute physical stress, it is necessary to examine whether similar changes occur under different types of physical stress.

This study aimed to clarify the variations in salivary HHV‐6 and HHV‐7 expression levels induced by different types of acute exercise, along with changes in conventional markers of physiological fatigue. We hypothesized that salivary HHV‐6 and HHV‐7 levels would increase to a greater extent during exercise that includes periods of higher intensity. To test this hypothesis, we performed using a crossover‐design experiment to investigate the effects of CE and IE on salivary HHV‐6 and HHV‐7 expression levels. Moreover, we examined whether these changes in HHV‐6 and HHV‐7 were associated with evaluations of subjective fatigue indexes, such as psychological questionnaires, and objective fatigue indexes, such as muscle strength.

## 2 MATERIALS AND METHODS

### 2.1 Ethical approval

All the participants voluntarily provided written informed consent before participating in the study. This study was approved by the Ethics Committee of Ritsumeikan University (BKC‐LSMH‐2019‐032) and was conducted in accordance with the Declaration of Helsinki.

### 2.2 Participants

Eleven healthy young men with no exercise habits (mean ± standard deviation [SD]: age 22 ± 1.1 years; height 171.6 ± 5.9 cm; body weight 65.6 ± 4.4 kg; $\dot{V}O$_2_max 44.3 ± 5.8 mL/kg/min [34.0–52.9]) participated in this study. All participants were recruited from Japan. Participants were excluded if, in the last year, they had physiological disorders or chronic diseases, were currently taking any medications, smoked or consumed alcohol daily, or had engaged in physical activity for 30 min or more at least twice per week in the past year.

### 2.3 Experimental procedures

After obtaining informed consent, $\overset{˙\;}{V}O$_2_max was measured approximately 1 week before the trials to determine the exercise intensity. The cycling exercise load for each trial was based on the $\dot{V}O$_2_max‐work rate relationship. Participants were instructed not to perform excessive exercise and not consume alcohol for 24 h before the $\dot{V}O$_2_max measurement and each exercise trial. Participants refrained from excessive exercise, alcohol consumption, and eating after 22:00 the night before attending the measurements from 8:00 am to 12:00 pm. The participants arrived at the laboratory at 8:00 am and were seated for 45 min before each exercise trial. All participants performed two exercise trials (continuous exercise trial and interval trial) with a randomized crossover design. A washout period of at least 3 days between trials was prepared as previously described (Allgrove et al., 2008). For the continuous exercise (CE) trials, cycling exercise at 70% $\dot{V}O$_2_max was performed for 20 min. For the interval exercise (IE) trials, the participants performed 5 sets of interval cycling exercises for 2 min at 50% $\dot{V}O$_2_max and 2 min at 90% $\dot{V}O$_2_max. After each exercise trial, the participants were allowed to rest in a sitting position for 60 min. The heart rate (HR) (WEP‐5204; Nihon Kohden, Tokyo, Japan) was measured every 1 min until the end of the exercise. Saliva and blood samples were collected before (pre), immediately after (post‐0 min), 30 min (post‐30 min), and 24 h (post‐24 h) after the end of exercise. Saliva samples were collected by chewing a piece of paraffin wax (B.S.A paraffin wax; B.S.A, Aichi, Japan) for 1 min at a frequency of 1 chew/sec. The saliva samples were centrifuged at 1500*g* for 15 min to remove human‐derived cells, and the supernatant was used for viral DNA extraction (Tamai et al., 2025). The samples were stored at −80°C until viral DNA extraction. The maximal voluntary contraction (MVC) was measured after saliva and blood sampling at each time point. The participants were instructed to fast and not drink anything until 60 min after the end of the exercise. The laboratory temperature was 22.7 ± 0.8°C.

### 2.4 Measurement of $\dot{V}O$ _2_max

$\dot{V}O$_2_max was measured using an incremental cycle exercise test based on monitoring breath‐by‐breath oxygen consumption (AE‐310SRD; Minato, Osaka, Japan), as previously described (Uchino et al., 2024). After warm‐up at 60 W cycling for 5 min, participants performed cycling at 60 W ± 30 W in increments of 15 W each minute until exhaustion. The participants were instructed to maintain a pedaling speed of at least 60 rpm. If at least three of the following four criteria were met, the test was considered valid: [I] plateau in VO_2_ with an increase in maximal power output, [II] HRmax of the age‐predicted maximum (220−age ± 5 beats/min), [III] maximal respiratory exchange ratio of ≧1.1, and [IV] an RPE of ≧18 (Borg, 1982; American College of Sports Medicine, 2000).

### 2.5 Measurements

#### 2.5.1 Body composition

Body weight (BW) was measured using a body composition analyzer (RD‐801, TANITA, Tokyo, Japan), and BW was measured to the nearest 0.1 kg. Height was measured using a height meter (WB‐510; TANITA, Tokyo, Japan) to the nearest 0.1 cm.

#### 2.5.2 Measurement of subjective fatigue

Subjective fatigue was assessed by visual analog scale (VAS). It measures the severity of fatigue before and after exercise, and participants were asked to rate their fatigue on a 10‐cm line. The participants were instructed to mark the fatigue they felt, from 0 cm for not fatigued to 10 cm for extremely fatigued. We calculated the sum of the time‐dependent changes in subjective fatigue.

#### 2.5.3 Maximum voluntary contraction

After saliva collection, maximum knee extension muscle strength was assessed as a measure of MVC. The participants were seated with their right legs locked into a custom‐made dynamometer (Takei Scientific Instruments Co., Ltd., Niigata, Japan), and the hip and knee joints were fixed at 90°, as previously described (Uchida et al., 2024). Participants performed a gradual increase in knee extension force from baseline to a maximum over 2–3 s, then maintained the maximal force for 2 s. The maximum value of the two measurements was used as a representative value. The participants performed leg extension at a low workload contraction 5 min before the warm‐up and familiarization measurements. We calculated the sum of the time‐dependent changes in MVC.

#### 2.5.4 Measurement of salivary HHV‐6 and HHV‐7

Salivary viral DNA was extracted from 200 μL of saliva using the QIAamp MinElute Virus Spin Kit (57704, Qiagen Inc., Hilden, Germany), according to the manufacturer's protocol. The expression of HHV‐6 and HHV‐7 DNA copies was analyzed using real‐time PCR with TaqMan Gene Expression assays as previously described (Aoki et al., 2016). Real‐time PCR was performed on a Prism 7500 Fast Sequence Detection System 2.2 (Applied Biosystems), and cycle threshold values were calculated using the system software. Amplifications were performed in a total volume containing TaqFast qPCR Master mix (4444557, Applied Biosystems, CA, USA), PCR forward primer (100 μM), PCR reverse primer (100 μM), TaqMan probe (10 μM), the viral DNA (10 ng/well), and PCR‐grade water. The following primers were used for the qPCR: HHV‐6 forward primer, 5′‐GACAATCACATGCCTGGATAATG‐3′; HV‐6 reverse primer, 5′‐TGTAAGCGTGTGGTAATGGACTAA‐3′; HHV‐6 probe, 5′‐FAM‐ AGCAGCTGGCGAAAAGTGCTGTGC‐TAMRA‐3′; HHV‐7 forward primer, 5′‐CGGAAG TCACTGGAGTAATGAC‐3′; HHV‐7 reverse primer, 5′‐CCAATCCTTCCGAAACCGAT‐3′; and HHV‐7 probe, 5′‐FAM‐CCTCGCAGATTGCTTGTTGGCCATG‐TAMRA‐3′. The following run method was utilized: 95°C for 20 s, followed by 50 cycles of 95°C for 3 s and 60°C for 30 s. The salivary HHV‐6/7 DNA levels were calculated as the total HHV‐6 and HHV‐7 copy numbers relative to the total saliva volume collected over 1 min; this value was expressed as copies/min (Tamai, Hiraoka, et al., 2022).

#### 2.5.5 Measurement of blood parameters

Blood lactate concentration in whole blood was measured using Lactate Pro2 (LT‐1730, Arkray, Tokyo, Japan).

The collected blood samples were centrifuged at 1500 *g* for 15 min, and the serum was used for the assay. The samples were stored at −80°C until the assays. IL‐6 concentration was measured using a high‐sensitivity IL‐6 ELISA kit (HS600C, R&D Systems, MN, USA). The absorbance was measured at 450 nm by microplate reader using an xMark microplate spectrophotometer (Bio‐Rad Laboratories, Hercules, CA, USA).

### 2.6 Statistical analyses

All values were expressed as mean ± SD. Statistical evaluations for each parameter were performed using a two‐way analysis of variance (ANOVA) for repeated measures (time × group). Salivary HHV‐6, salivary HHV‐7, subjective fatigue, MVC, blood lactate, and change in IL‐6 concentrations were compared using 2 trials × 4 time points (pre, post‐0 min, post‐30 min, and post‐24 h) using two‐way repeated‐measures ANOVA. Bonferroni post hoc comparison test was performed to correct for multiple comparisons when the analyses revealed significant differences. Salivary HHV‐6 and HHV‐7 levels, subjective fatigue, lactate concentration, and IL‐6 concentration were non‐normally distributed. The MVC was normally distributed. Salivary HHV‐6, HHV‐7, and lactate concentrations were transformed into logarithms, and a two‐way ANOVA was performed. Changes in salivary HHV‐7 levels and subjective fatigue were compared using a paired *t*‐test. Changes in salivary HHV‐6 AUC and the sum of time‐dependent changes in MVC were compared using the Wilcoxon test. The relationships between changes in salivary HHV‐6 and changes in MVC, blood lactate, subjective fatigue, and IL‐6 concentration were determined using Spearman's rank correlation coefficients. Statistical significance was defined as a *p* value <0.05. All statistical analyses were performed using the IBM SPSS software (version 29.0; International Business Machines Corp., USA). The statistical power for the log salivary HHV‐6 outcomes in this study was calculated post hoc using the G*Power software (version 3.1.9.6), with an effect size of 0.543 and a power of 0.98 for two‐way repeated measures ANOVA.

## 3 RESULTS

### 3.1 Change in subjective fatigue and muscle strength

Significant main effects of time were observed for subjective fatigue (*p* < 0.001), fold change in subjective fatigue (*p* = 0.006), changes in subjective fatigue (*p* < 0.001), MVC (*p* = 0.003), fold change in MVC (*p* = 0.005), and change in MVC (*p* = 0.003). Significant main effects of the trial were observed for the fold‐change in MVC (*p* = 0.042) and change in MVC (*p* = 0.032). However, no significant interactions were observed.

**FIGURE 1:**
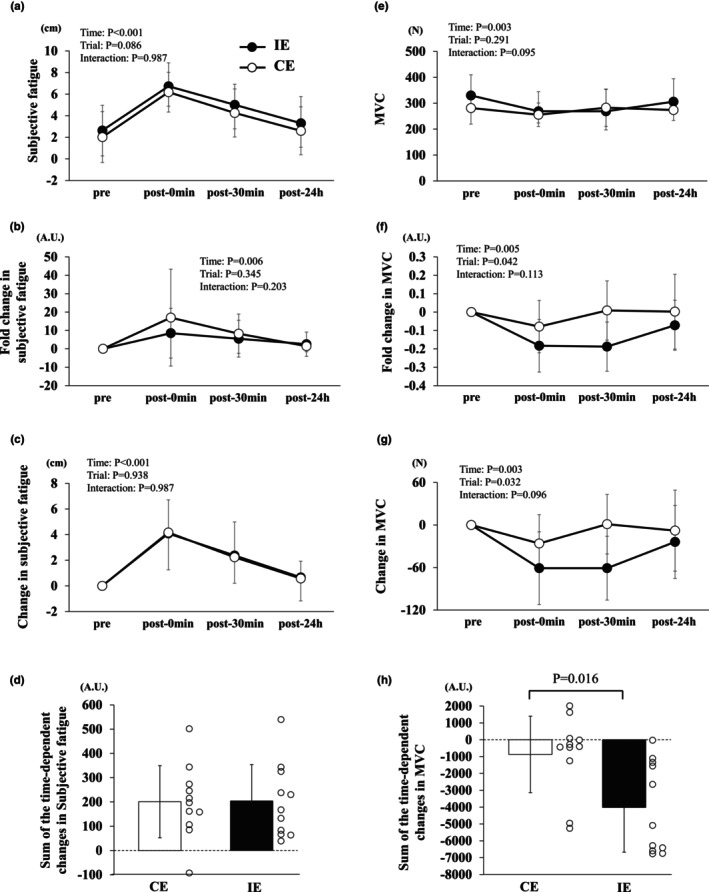
Effects of CE and IE on subjective fatigue and MVC (a, e), fold change in subjective fatigue and MVC (b, f), change in subjective fatigue and MVC (c, g), sum of the time‐dependent changes in subjective fatigue and MVC (d, h) in healthy untrained young men (*n* = 11). Data are expressed as mean ± SD.

No significant differences were observed between the trials in subjective fatigue, fold change in subjective fatigue, and changes in subjective fatigue (Figure 1a–c). No significant differences in the sum of the time‐dependent changes in subjective fatigue from pre to post‐24 h were observed among the trials (Figure 1d). No significant differences were observed between the trials in MVC, fold change in MVC, and changes in MVC (Figure 1e–g). However, the sum of the time‐dependent changes in MVC in IE was significantly lower than that in CE (*p* = 0.016, Figure 1h).

### 3.2 Changes in salivary HHV‐6 and HHV‐7

Significant main effects of time were observed for log salivary HHV‐6 (*p* < 0.001), fold change in salivary HHV‐6 (*p* = 0.020), changes in salivary HHV‐6 (*p* = 0.006), salivary HHV‐7 (*p* = 0.002), and changes in salivary HHV‐7 (*p* = 0.003). Significant effects of the trial were observed for log salivary HHV‐6 (*p* = 0.038) and changes in salivary HHV‐6 (*p* = 0.041). A significant interaction was observed between salivary HHV‐6 (*p* = 0.006), fold‐change in salivary HHV‐6 (*p* = 0.039), and changes in salivary HHV‐6 (*p* = 0.048).

**FIGURE 2:**
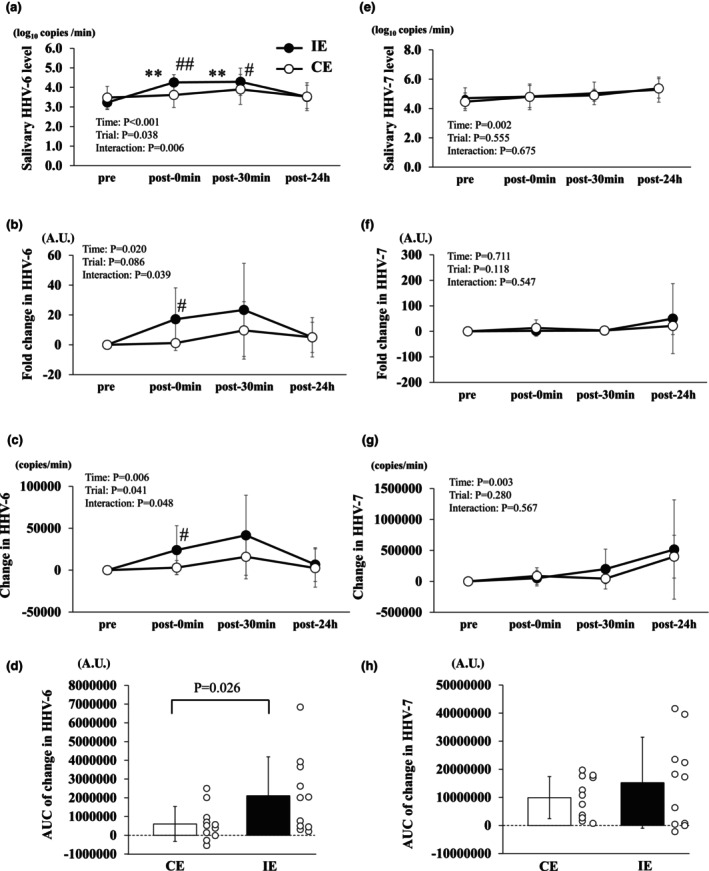
Effects of CE and IE on the salivary HHV‐6 and HHV‐7 levels (a, e), fold change in salivary HHV‐6 and HHV‐7 (b, f), change in salivary HHV‐6 and HHV‐7 (c, g), and salivary HHV‐6 and HHV‐7 AUC (d, h) in healthy untrained young men (*n* = 11). The salivary HHV‐6 and HHV‐7 levels (a, e) were calculated as the total HHV‐6 and HHV‐7 copy numbers relative to the total saliva volume collected over 1 min. Data are expressed as mean ± SD. *p < 0.05 versus pre in each trial, ^#^ *p* < 0.05 versus CE at the same time point, t *p* < 0.05 versus at post‐0 min in each trial.

Salivary HHV‐6 expression level in the IE trial increased at post‐0 min (*p* < 0.001) and post‐30 min (*p* = 0.002) compared to pre (Figure 2a). Salivary HHV‐6 expression level was significantly higher in the IE trial than in the CE trial at post‐0 min (*p* = 0.002) and post‐30 min (*p* = 0.0048, Figure 2a). Salivary HHV‐6 expression levels in the CE trial did not change post‐30 min compared to pre (Figure 2a). Fold change in salivary HHV‐6 and changes in salivary HHV‐6 expression levels were significantly higher in the IE trial than in the CE trial at post‐0 min (*p* = 0.029, *p* = 0.011 respectively; Figure 2b,c). The AUC of change in salivary HHV‐6 in IE was significantly higher than in CE (*p* = 0.026, Figure 2d). No significant differences were observed between the trials in salivary HHV‐7, fold change in salivary HHV‐7, or changes in salivary HHV‐7 (Figure 2e–g). The AUC of the change in HHV‐7 levels from pre to post‐24 h showed no significant differences among the trials (Figure 2h).

### 3.3 Changes in circulating lactate and IL‐6 levels

Significant effects of time were noted with respect to blood lactate levels (*p* < 0.001) and changes in serum IL‐6 concentrations (*p* < 0.001). Additionally, significant main effects of the trial were identified for blood lactate levels (*p* < 0.001) and alterations in serum IL‐6 concentrations (*p* = 0.012). Furthermore, a significant interaction was observed between blood lactate levels (*p* < 0.001) and changes in serum IL‐6 concentrations (*p* = 0.022).

**FIGURE 3:**
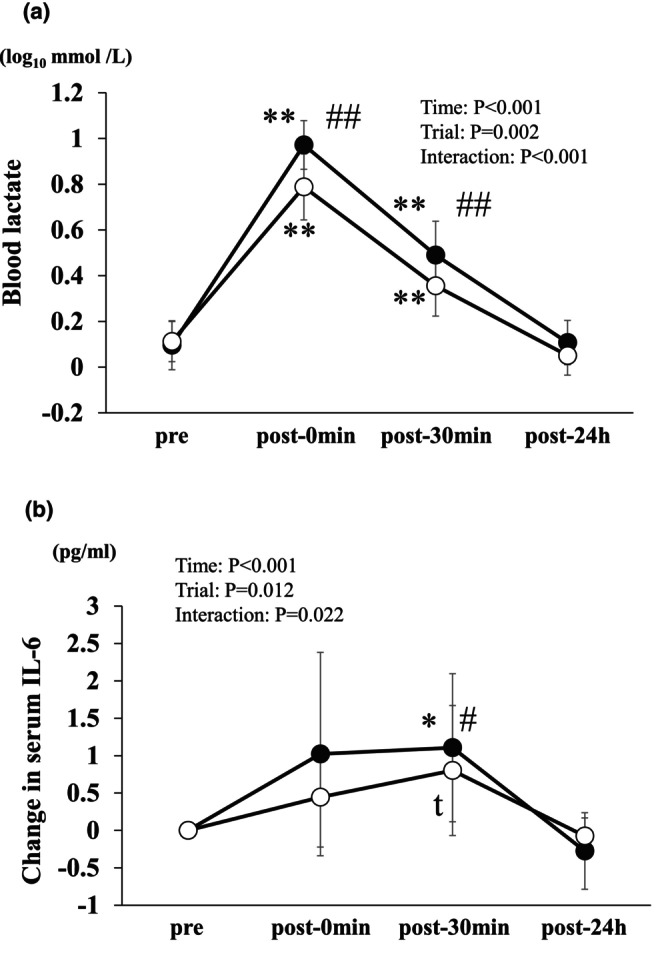
Effects of CE and IE on blood lactate (a) and change in serum IL‐6 (b) in healthy untrained young men (*n* = 11). Data are expressed as mean ± SD. *p < 0.05 versus pre in each trial. ^#^ *p* < 0.05 versus CE at the same time point.

Blood lactate concentration increased post‐0 min (*p* < 0.001) and post‐30 min (*p* < 0.001) in the IE trial (Figure 3a). Blood lactate concentration increased post‐0 min (*p* < 0.001) and post‐30 min (*p* = 0.008) in the CE trial (Figure 3a). The blood lactate concentration at post‐0 min (*p* < 0.001) and post‐30 min (*p* = 0.001) was significantly higher in the IE trial than in the CE trial (Figure 3a). Changes in serum IL‐6 concentration increased at post‐30 min in the IE trial (*p* = 0.024, Figure 3b). Changes in serum IL‐6 concentration in the CE trial increased at post‐30 min compared to post‐0 min (*p* = 0.006, Figure 3b). Changes in serum IL‐6 concentration at post‐30 min in the IE trial were significantly higher than those in the CE trial (*p* = 0.040, Figure 3b).

### 3.4 Correlation between change in salivary HHV‐6 and changes in other parameters

Changes in salivary HHV‐6 levels from pre to post‐24 h were significantly positively correlated with changes in blood lactate and IL‐6 concentrations in the IE trial (Table 1; *r*_s_ = 0.597, *p* < 0.001; *r*_s_ = 0.553, *p* < 0.001, respectively). Changes in salivary HHV‐6 levels from pre to post‐24 h were significantly negatively correlated with changes in MVC in the IE trial (Table 1; *r*_s_ = −0.349, *p* = 0.047). However, no significant correlation between the changes in salivary HHV‐6 levels and changes in subjective fatigue was observed in the IE trial.

**TABLE 1:** Correlations between change in salivary HHV‐6 expression levels and change in blood lactate, subjective fatigue, IL‐6 or MVC in IE trial.

	Δ HHV‐6 (copies/min)	
	*r*_s_	*p*
Δ Blood lactate (mmol/L)	0.597	<0.001
Δ IL‐6 (pg/mL)	0.553	<0.001
Δ Subjective fatigue (cm)	0.313	0.077
Δ MVC (N)	−0.349	0.047

Abbreviations: HHV‐6, human herpes virus‐6; MVC, maximum voluntary contraction; *r*_s_, Spearman's rank correlation coefficient.

## 4 DISCUSSION

In this study, the salivary HHV‐6 expression increased after acute exercise in the IE trial and returned to baseline after 24 h. This increase in salivary HHV‐6 expression after the IE trial was significantly higher than after the CE trial and did not change in the CE trial. Additionally, the change in salivary HHV‐7 expression did not differ after exercise in the IE and CE trials. Thus, IE may be an exercise pattern in which salivary HHV‐6 expression increases more than in CE. Furthermore, in this study, the increase in subjective fatigue after exercise did not differ between the two trials. However, the sum of the time‐dependent changes in MVC after exercise was significantly lower in the IE trial than in the CE trial. Additionally, changes in salivary HHV‐6 expression were negatively associated with changes in MVC in the IE trial. These findings suggest that increased salivary HHV‐6 levels may reflect objective physical fatigue after exercise.

Previous studies have shown that salivary HHV‐6 expression increases during high‐intensity field training in military individuals (Aoki et al., 2016), for example, during a 3‐week training camp in university baseball and rugby players (Tamai, Sone, et al., 2022), and during a 3‐day judo training camp (Tamai, Hiraoka, et al., 2022). However, the effects of a single bout of acute exercise on salivary HHV‐6 and ‐7 expression remain unclear. This study examined, for the first time, fluctuations in salivary HHV‐6 levels after transient exercise. The findings showed that salivary HHV‐6 in the IE trial significantly increased after exercise but did not significantly change in the CE trial. The mechanism underlying changes in HHV‐6 expression in response to acute exercise remains unclear. Several studies have shown activation of glycolytic metabolism is involved in the reactivation of HHV‐6 (Ma et al., 2023; Wu et al., 2020). In this study, blood lactate levels, the augmented metabolic product of glycolysis, were significantly higher in the IE trial than in the CE trial. Moreover, changes in salivary HHV‐6 expression were positively associated with changes in blood lactate levels in the IE trial. Therefore, these findings suggest that activation of glycolytic metabolism in the IE trial may induce an increase in salivary HHV‐6 expression, resulting in different responses to the CE trial with less glycolytic activation. Additionally, inflammatory cytokines such as IL‐6 lead to reactivation of HHV‐6 (Fujita et al., 2008; Yoshikawa et al., 2006). In patients with drug‐induced hypersensitivity syndrome, HHV‐6 reactivation and increased IL‐6 levels have been observed (Yoshikawa et al., 2006). In this study, the circulating levels of IL‐6 were significantly higher in the IE trial than in the CE trial. Furthermore, changes in salivary HHV‐6 expression are positively associated with changes in circulating IL‐6 levels in the IE trial. Therefore, these findings suggest that the increase in inflammatory cytokines, such as IL‐6, in the IE trial may induce an increase in salivary HHV‐6 expression, and the difference in the inflammatory response to exercise patterns may be involved in the difference in response to HHV‐6 expression between IE and CE trials. Lactate has been shown to be associated with IL‐6 production (Cullen et al., 2016), thus further investigation is required to elucidate the effect of the interaction between IL‐6 and lactate on the increase in salivary HHV‐6 levels.

In this study, subjective fatigue significantly increased after exercise in both trials; however, no significant differences were observed between the two trials. In contrast, the sum of the time‐dependent changes in MVC in the IE trial was significantly lower than that in the CE trial, suggesting that MVC decreased more after exercise, and recovery was delayed during the post‐exercise period in the IE trial. Therefore, these results suggest that the physical fatigue state occurring in vivo, although not subjectively detected, may be higher in IE than in CE. In fact, changes in salivary HHV‐6 expression were negatively associated with changes in MVC in the IE trial. Previous studies have shown that subjective fatigue measured using the Chalder Fatigue Scale does not correlate with salivary HHV‐6 expression (Aoki et al., 2016). Furthermore, in a cross‐sectional study, caregivers with frequent night shifts that induce physical fatigue showed increased salivary HHV‐6 expression (Aoki et al., 2016). These findings suggest that salivary HHV‐6 levels may be associated with transient fatigue caused by physical loads such as high‐intensity exercise.

In this study, salivary HHV‐7 levels did not increase after exercise, and no differences were found between the two trials. A previous study has shown that salivary HHV‐7 expression increases with subjective fatigue during high‐intensity field training in military individuals (Aoki et al., 2016). Furthermore, Aoki et al. (2016) discussed the possibility that salivary HHV‐7 may reflect longer‐term fatigue than HHV‐6. Therefore, it may be necessary to examine the influence of long‐term exercise to clearly observe changes in salivary HHV‐7 expression levels.

This study had a few limitations. First, acute psychological stress may affect the immune system (Cain & Cidlowski, 2017), leading to the reactivation of HHV‐6. Further studies need to examine the effect of HHV‐6 on psychological stress to better disentangle physical and psychological contributions. Second, circulating lactate is associated with IL‐6 production (Cullen et al., 2016); thus, further studies are required to elucidate the effect of the interaction between IL‐6 and lactate on the increase in salivary HHV‐6 levels. Third, heart rate response is a convenient and easily measurable indicator of physiological stress induced by exercise. Thus, further studies are needed to compare HHV‐6 variability with heart rate response as a marker reflecting physical fatigue. Fourth, further studies with larger sample sizes are required to investigate the effects of sex and other participant characteristics on salivary HHV‐6 variability after exercise.

To our knowledge, this study is the first to report fluctuations in salivary HHV‐6 levels in response to acute exercise. Furthermore, an increase in salivary HHV‐6 levels was associated with a decrease in MVC, a muscle fatigue indicator. These findings provide evidence that may contribute to the appropriate management of daily exercise training in athletes and physically active individuals. Additionally, further evaluation of the reliability and stability of salivary HHV‐6 fluctuations, in comparison with other noninvasive indicators such as heart rate and blood pressure, could establish HHV‐6 as a promising novel noninvasive conditioning marker for athletes and trained populations. Further investigation is warranted to determine whether salivary HHV‐6 variability can be used as a biomarker for evaluating training status across different athletic sports by examining how salivary HHV‐6 responds to various types of training.

In summary, the results of this study suggest that IE may increase salivary HHV‐6 expression, and this increase may be associated with glycolytic metabolism and inflammatory cytokines. Furthermore, as salivary HHV‐6 expression correlates with a decrease in MVC after IE, changes in salivary HHV‐6 expression may reflect objective physical fatigue after acute IE.

## AUTHOR CONTRIBUTIONS

M.U. and M.I. conceived and designed the research; M.U. and M.I. performed experiments; M.U. analyzed data; all authors interpreted results of experiments; M.U. and M.I. prepared figures; T.I. and M.I. acquired the research funding; M.U., C.K., T.H., and M.I. drafted the manuscript; all authors edited and revised the manuscript; and all authors approved the final version of the manuscript.

## FUNDING INFORMATION

This work was supported by Grants‐in‐Aid for Scientific Research from the Ministry of Education, Culture, Sports, Science, and Technology of Japan (KAKENHI: 22H03487 for M. Iemitsu) and the Leading‐edge Research Project for Sports Medicine and Science of the Japan Sports Agency (to T. Isaka). This study was funded by Ritsumeikan Global Innovation Research Organization (M. Iemitsu).

## CONFLICT OF INTEREST STATEMENT

No conflicts of interest, financial or otherwise, are declared by the authors.

## Data Availability

The data supporting the findings of this study is available from the corresponding author upon request.
